# Prevalence and Characterization of Shiga Toxin-Producing and Enteropathogenic *Escherichia coli* in Shellfish-Harvesting Areas and Their Watersheds

**DOI:** 10.3389/fmicb.2015.01356

**Published:** 2015-12-01

**Authors:** Charlotte Balière, Alain Rincé, Jorge Blanco, Ghizlane Dahbi, Josée Harel, Philippe Vogeleer, Jean-Christophe Giard, Patricia Mariani-Kurkdjian, Michèle Gourmelon

**Affiliations:** ^1^Laboratoire Santé Environnement et Microbiologie, Unité Santé, Génétique et Microbiologie des Mollusques, Département Ressources Biologiques et Environnement, IfremerPlouzané, France; ^2^U2RM EA4655 Stress/Virulence, Normandie-Université, University of Caen NormandyCaen, France; ^3^Departamento de Microbioloxía e Parasitoloxía, Facultade de Veterinaria, Universidade de Santiago de CompostelaLugo, Spain; ^4^Groupe de Recherche sur les Maladies Infectieuses du Porc, Département de Pathologie et Microbiologie, Faculté de Médecine Vétérinaire, Centre de Recherche d’Infectiologie Porcine et Avicole, Université de MontréalSaint-Hyacinthe, QC, Canada; ^5^U2RM EA4655 Antibio-Résistance, Normandie-Université, University of Caen NormandyCaen, France; ^6^Service de Microbiologie, CNR Associé Escherichia coli, AP-HP, Hôpital Robert-DebréParis, France; ^7^Infection, Antimicrobials, Modelling, Evolution, UMR 1137, INSERMParis, France; ^8^Infection, Antimicrobials, Modelling, Evolution, UMR 1137, Université Paris Diderot – Sorbonne Paris CitéParis, France

**Keywords:** STEC, EPEC, shellfish, water, MLST, PFGE, biofilms

## Abstract

more strains formed a strong biofilm at 18 than at 30°C. Finally, more than 85% of analyzed strains were found to be sensitive to the 16 tested antibiotics. These data suggest the low risk of human infection by STEC if shellfish from these shellfish-harvesting areas were consumed.

## Introduction

The microbiological quality of coastal environments can be impacted by urban and agricultural fecal wastes from watersheds. Moreover, shellfish can accumulate and concentrate pathogenic micro-organisms, such as *Salmonella*, pathogenic *Escherichia coli* (*E. coli*) and noroviruses present in surrounding waters by their filter-feeding activities (Potasman et al., 2002). This can lead to closures or downgrading of shellfish-harvesting areas and to outbreaks of food poisoning through consumption of contaminated shellfish (Iwamoto et al., 2010).

Enumeration of *E. coli*, a fecal bacterial indicator, is the standard way to assess the level of fecal microorganisms in water and shellfish and indirectly, to estimate the associated potential risk to human health from all waterborne enteric pathogens (e.g., through classification of bathing areas and shellfish-harvesting areas; Anonymous, 2004). However, in addition to being a fecal indicator and a commensal bacterium, *E. coli* includes strains that can be pathogenic to humans. These can cause diarrhea and extra-intestinal diseases after acquiring virulence genes by genetic mobile elements such as bacteriophages, pathogenicity islands, and plasmids (Touchon et al., 2009). Pathogenic *E. coli* are distributed into diarrheagenic *E. coli* pathotypes including enterotoxigenic *E. coli* (ETEC), *Shigella*/enteroinvasive *E. coli* (EIEC), enteroaggregative *E. coli* (EAEC), diffusely adherent *E. coli* (DAEC), enteropathogenic *E. coli* (EPEC), Shiga toxin-producing *E. coli* (STEC; for review, Croxen et al., 2013) and into extra-intestinal *E. coli* pathotypes (Russo and Johnson, 2000).

*Escherichia coli* occurrence in seafood is considered a sanitary case and may represent a risk to the consumers if related to diarrheagenic *E. coli* (for review, Costa, 2013).

The study presented here focuses on EPEC (one of the main causes of diarrhea in infants) and STEC (an emerging zoonotic pathogen).

Enteropathogenic *E. coli* is an important cause of infantile watery diarrhea, which is more frequently encountered in low-income countries than in the industrialized world (Nataro and Kaper, 1998). They are known to create distinctive lesions on the surface of intestinal epithelial cells, called attaching and effacing (A/E) lesions. This property is encoded by genes, including *eae*, grouped together in a pathogenicity island referred to the ‘locus of enterocyte effacement’ (LEE; Paton and Paton, 1998). EPEC is transmitted from host to host via the fecal-oral route through contaminated surfaces, waters and food and human carriers. Humans, including symptomatic and asymptomatic children and asymptomatic adults, are the most likely source (Levine and Edelman, 1984). Animals, such as cattle and wildlife species, have been found to be additional sources (Singh et al., 2015). Twelve O serogroups have been recognized as EPEC by the World Health Organization: O26, O55, O86, O111, O114, O119, O125, O126, O127, O128, O142, and O158 (WHO, 1987).

Shiga-toxin producing *Escherichia coli* are responsible for the mucoid-bloody diarrhea that can progress to hemolytic uremic-syndrome (HUS), especially in children. One of the most important pathogenicity factors produced by STEC strains is the Shiga toxin (Stx), encoded by a lambdoid bacteriophage (O’Brien et al., 1984). Shiga toxins can be divided into two types, Stx1 (almost identical to Shiga toxin produced by *Shigella dysenteriae* type 1) and Stx2, encoded by *stx1* and *stx2* genes, respectively (Scheutz et al., 2012). In addition, the STEC strains are often able to produce the A/E lesions as a result of the presence of the LEE pathogenicity island, as in EPEC. This subset of STEC strains is also known as enterohemorrhagic *E. coli* (EHEC; McDaniel et al., 1995). Instead of this LEE pathogenicity island, they can also possess the auto-agglutinating adhesin factor designated Saa (STEC autoagglutinating adhesin; Paton et al., 2001). Adhesion to the intestinal mucosa is an essential step in the infection cycle of *E. coli*, which contributes to pathogenesis in humans. Other factors are involved in the virulence of STEC but also of EPEC, such as enterohemolysin A, encoded by the *ehxA* gene and associated with cytotoxic effects on endothelial cells that may contribute to the development of HUS (Jiang et al., 2015). STEC infections have been reported following the ingestion of contaminated food or water, after bathing in contaminated waters or contact with animals (for review, Croxen et al., 2013). The principal reservoir of STEC is the digestive tract of animals, particularly of cattle that are healthy carriers (Bibbal et al., 2015). Other animals, such as sheep, goats, swine, birds, and other wild animals, as well as humans, can also harbor STEC (Mora et al., 2012; Chandran and Mazumder, 2013).

Most human illness is caused by the serotype STEC O157:H7 (Paton and Paton, 1998). However, it is becoming evident that non-O157 isolates belonging to the serogroups O26, O45, O91, O103, O111, O113, O121, O145 also cause significant human illness (Mellmann et al., 2009; USDA, 2011). In Europe, O157:H7 and the four serotypes: O26:H11, O103:H2, O111:H8, and O145:H28 are the most widely implicated in human STEC infections, constituting the five highly pathogenic serotypes (EFSA, 2013).

Shiga-toxin producing *Escherichia coli* and EPEC contamination of the environment may occur through the spreading of livestock manure, animal waste on pastures, via wastewaters from slaughterhouses or from treatment plant eﬄuents and by wildlife (Muniesa et al., 2006; Singh et al., 2015).

In such environments, STEC and EPEC strains are exposed to various stresses, such as low temperature or nutrient depletion and the ability to form biofilm could be an advantage to increase persistence (Vogeleer et al., 2014).

To date, very few studies have focused on the detection and isolation of pathogenic *E. coli* belonging to the STEC and EPEC pathovars in coastal environments (Gourmelon et al., 2006; Bennani et al., 2011). The aim of the study presented here was to detect and characterize STEC and EPEC strains from French shellfish-harvesting areas and their upstream watershed in order to assess the diversity of these pathogenic *E. coli* potentially present in this type of hostile environment. For this purpose, during a 2-years study, shellfish batches, freshwater, seawater, and surface sediment samples from three selected shellfish-harvesting areas and their upstream watersheds, the location of intensive livestock activities (cattle, swine, poultry, and/or sheep), were analyzed monthly to evaluate the presence of STEC and EPEC strains.

## Materials and Methods

### Sampling Locations and Sample Description

Shellfish, water, and surface sediment samples were collected from three shellfish-harvesting sites on the French coast of within the Eastern English Channel and their watersheds. One of these sites, located in Brittany (site 1), corresponded to a 121 km^2^ watershed, characterized by intensive livestock farming (cattle, swine, and poultry), with a human population of about 9,000 inhabitants. The two others were situated in Normandy; site 2 was characterized by a 1,000 km^2^ catchment, with large livestock farming (cattle, sheep, swine, and poultry) and about 40,000 inhabitants, while the second site (site 3) corresponded to a 50 km^2^ watersheds with large livestock farming (cattle, sheep, and swine) and about 7,000 inhabitants. These two latter watersheds are geographically closer together than the Brittany site (location of the sites Supplementary Figure S1). The three shellfish-harvesting areas are classified as category B for oysters (*Crassostrea gigas*) and mussels (*Mytilus edulis*) and as category C for common cockles (*Cerastoderma edule*) according to European regulation (European Directive 91/492/EEC; Anonymous, 2004). Shellfish from category B shellfish-harvesting areas must be depurated before being sold and shellfish from category C areas must be relayed at least 2 months prior to sale for consumption. Shellfish [oyster, mussel, and common cockle batches (site 1, *n* = 120; site 2, *n* = 72; and site 3, *n* = 46)] and freshwater samples from nine sampling sites upstream of shellfish-harvesting areas (site 1, *n* = 96; site 2, *n* = 72; site 3, *n* = 48) were collected monthly from February 2013 to January 2015, whereas surface sediment samples (site 1, *n* = 13; site 2, *n* = 13; site 3, *n* = 13) were collected from February 2013 to January 2014 and seawater samples (site 1, *n* = 12) from February 2014 to January 2015.

### Isolation of STEC and EPEC Strains

Samples were transported in insulated cooler boxes to the laboratory and analyzed within 24 h. After opening, total shellfish flesh, including shellfish flesh and intravalvular liquid, were homogenized in a commercial blender (Waring Products Division, Torrington, CT, USA) for 60 s at high speed. Twenty-five grams of homogenized total shellfish flesh were inoculated into 225 ml of buffered peptone water (BPW). For surface sediments, 10 g were introduced into the same volume of BPW. For water samples, 1 L was filtered using 0.45 μm cellulose membranes (Pall Gelman GN-6 Metricel; Pall Corporation, St Germain-en-Laye, France) and the filter was placed in 225 ml of BPW. Incubation was performed at 37°C for 24 h.

Shiga-toxin producing *Escherichia coli* and EPEC strains were isolated from the environmental samples using three additional protocols. The first one, described by Balière et al. (2015), involves application of the ISO/TS-13136 method, which focuses on isolation of strains belonging to the five highly pathogenic serotypes and was applied to samples collected from February 2013 to February 2014. The two other protocols involve the isolation of STEC and EPEC with or without an enrichment step and independently from the serotype. These were applied to samples collected from February 2013 to January 2015.

For the protocol with an enrichment step (described in Balière et al., 2015), DNA was extracted from 500 μL of each BPW enrichment broth using NucliSENS Nucleic Acid Extraction Reagents for miniMAG (BioMérieux, Marcy l’Etoile, France), according to the manufacturer’s instructions. The *stx* and *eae* genes were detected by real-time PCR (Agilent MX3000P, Waldbronn, Germany), using primers and probes published previously (Nielsen and Andersen, 2003; Perelle et al., 2007), according to the ISO/TS-13136: 2012 technical specification, with slight modifications concerning the PCR cycles [denaturation for 10 s at 95°C, primer annealing for 5 s at 55°C, and extension for 25 s at 60°C (45 cycles)]. BPW broths identified positive for *stx* and *eae* were screened for STEC and EPEC isolates by streaking 1 μL of these broths onto Tryptone-Bile-X-glucuronide agar (TBX; AES chemunex, Bruz, France) and onto chromID^TM^ agar (BioMérieux), followed by incubation at 44°C for 24 h.

The final protocol to be used involves the screening of *E. coli* isolated directly from the water and shellfish samples without an enrichment step. For this protocol, 1, 10, and 100 ml of water were filtered through 0.45 μm cellulose membranes and the filters were placed onto TBX agar. For shellfish, 10 g of blended total shellfish flesh were distributed onto five empty and sterile plates with overlay super-cooled TBX agar. All TBX plates were incubated at 44°C for 24 h. Presumptive STEC and EPEC isolates were confirmed by real-time PCRs targeting *stx1*, *stx2*, and *eae* genes, as described above after a DNA extraction of each isolate by boiling at 100°C, for 15 min.

The STEC and EPEC isolates were characterized using several protocols as described below.

### Serotyping

The serotypes of the STEC and EPEC strains were characterized using the serotyping method by agglutination, as described by Blanco et al. (2003).

More precisely, determination of O and H antigens was carried out by agglutination as previously described (Guinée et al., 1981), employing all available O (O1-O185) and H (H1-H56) antisera. All antisera were absorbed with the corresponding cross-reacting antigens to remove the non-specific agglutinins. The O and H antisera were produced in the Laboratorio de Referencia de *E. coli* (USC, Lugo, Spain). Isolates that did not react with O antisera were considered as non-typeable (ONT) and those non-motile were HNM.

### Detection of Enterohemolysin and Adhesin

The presence of e*hxA* (encoding enterohemolysin A) and *saa* (encoding STEC autoagglutining adhesin) genes in these isolates was investigated by conventional PCR using primers previously described by Paton and Paton (2002).

### Phylogenetic Group

Isolates were classified into the four main *E. coli* phylogenetic groups (A, B1, B2, or D) using a conventional triplex PCR method based on the detection of two genes, *chuA*, required for heme transport in enterohemorrhagic O157:H7 *E. coli, yjaA*, initially identified in the recent complete genome sequence of *E. coli* K-12, for which the function is unknown, and of an anonymous DNA fragment designated TSPE4.C2 using primers described previously by Clermont et al. (2000).

### Pulsed-field Gel Electrophoresis

The genetic relatedness of the isolates was studied by the pulsed-field gel electrophoresis method (PFGE) according to Bidet et al. (2005). Isolated strains were inoculated in nutrient broth containing 1.3% NaCl (Bio-Rad, Marnes-la-coquette, France) and incubated at 37°C for 24 h. Bacterial DNA was extracted from 400 μl of the enrichment broth using the CHEF Bacterial Genomic DNA Plug Kit (Bio-Rad) according to the manufacturer’s recommendations. Bacterial DNA was digested for between 16 and 20 h at 37°C with the restriction endonuclease *XbaI* (Roche Diagnostic, Meylan, France) according to the manufacturer’s recommendations. Each electrophoresis was performed using a lambda ladder molecular mass marker (Bio-Rad) for the normalization of gel images. The migration was performed on a 1% agarose gel using the CHEF-DRIII apparatus (Bio-Rad) at 6 V cm^-1^ for 27 h, with pulse times varying linearly between two and 49 s. The bacterial DNA restriction patterns were analyzed using the Bionumerics software 7.5 (Applied Maths, Kortrijk, Belgium). The similarity of PFGE profiles was compared and a dendrogram was created using the band-based Dice unweighted-pair group method, using average linkages (UPGMA), based on 1% position tolerance and 0% position optimization. Branch quality was evaluated using Cophenetic correlation. PFGE patterns were considered clonally related when they had a similarity coefficient higher than 80%.

### Multilocus Sequencing Typing

The genetic relatedness of the isolates was also studied using the multilocus sequencing typing method (MLST). Fragments of seven housekeeping genes, i.e., *adk* (adenylate kinase), *fumC* (fumarate hydratase), *gyrB* (DNA gyrase), *icd* (isocitrate/isopropylmalate dehydrogenase), *mdh* (malate dehydrogenase), *purA* (adenylosuccinate dehydrogenase), and *recA* (ATP/GTP binding motif) were amplified and sequenced using suitable primers (Wirth et al., 2006) with minor modifications for the *recA* primers (recAR 5′-TCG-TCG-AAA-TCT-ACG-GAC-CGG-A-3′; recAF1 5′-ACC-TTT-GTA-GCT-GTA-CCA-CG-3′). The PCR cycle included denaturation for 60 s at 95°C, primer annealing for 60 s at 56°C (for *adk*, *purA, recA*, and *icd*), at 65°C (for *mdh* and *gyrB*), or at 68°C (for *fumC*), and extension for 60 s at 72°C (35 cycles) in MJ Research PTC-200 (DNA Engine, Waltham, MA, USA). Sequencing was performed in both directions with the fluorescent dye terminator Sanger method on ABI3730 (Applied Biosystem) by Eurofins Genomics (Ebersberg, Germany). The alleles and sequence types (ST) were assigned in accordance with the *E. coli* MLST database (http://mlst.warwick.ac.uk/mlst/dbs/Ecoli).

### Static Biofilm Formation Assay

A biofilm formation assay was performed as previously described by Tremblay et al. (2015). In addition to the incubation temperature of 30°C, the ability to form biofilms was also tested at 18°C, in order to reproduce marine temperate environmental conditions (Moldoveanu, 2012). Briefly, overnight cultures at 37°C in LB media were diluted (1:100) in 5 ml of M9 medium with glucose (0.4% wt/vol) and minerals (1.16 mM MgSO_4_, 2 μM FeCl_3_, 8 μM CaCl_2_, and 16 μM MnCl_2_) and incubated for 24 h at 37°C. These cultures were diluted (1:100) in M9 medium supplemented with glucose and minerals and were inoculated in triplicate into microtitre plates (Costar 3370; Corning, NY, USA). After 24 h of incubation at 18 or 30°C, unattached cells were removed by washing three times with phosphate-buffered saline (PBS). Plates were dried at 37°C for 15 min and biofilms were stained with crystal violet (0.1% wt/vol) for 2 min. After removal of crystal violet solution, the biofilms were washed three times with PBS and dried at 37°C for 15 min. The biofilm stain was dissolved with 150 μl of 80% (vol/vol) ethanol and 20% (vol/vol) acetone and biofilms were quantified by measuring the absorbance at 590 nm (OD_590_) with a microplate reader (Powerwave; BioTek Instruments, Winooski, VT, USA). The results for the static biofilms formed at 18 and 30°C were compared using two-way analysis of variance (ANOVA) followed by a Bonferroni *post hoc* comparison using GraphPad Prism, version 4.02 (GraphPad Software, San Diego, CA, USA). Strains were divided into three groups based on the OD_590_ of bacterial biofilm: strong (A_590_ > 0.6), medium (0.6 ≥ A_590_ ≥ 0.3) and weak or none (A_590_ < 0.3).

### Antibiotic Resistance

Antimicrobial susceptibility testing based on the disk diffusion method was performed on a selection of STEC and EPEC isolates. Sixteen antibiotics were tested: Tobramycin (10 μg), Fosfomycin (50 μg), Cefalotin (30 μg), Imipenem (10 μg), Tigecyclin (15 μg), Gentamycin (15 μg), Cefotaxim (30 μg), Cefoxitin (30 μg), Doxycyclin (30 μg), Ciprofloxacin (5 μg), Augmentin (20 μg amoxicilin; 10 μg clavulanic acid), Ticarcillin (75 μg), Bactrim (1.25 μg trimethoprim; 23.75 μg sulfamethoxazol), Nalidixic acid (30 μg), Amikacin (30 μg), Amoxicillin (25 μg) on Mueller-Hinton medium (AES chemunex, Bruz, France). Plates were incubated at 37°C for 24 h.

### Environmental Data and Statistical Analysis

Rainfall data (2-days cumulative rainfall before sampling date) were provided by the meteorological stations from Meteo France at Pleurtuit (site 1) and at Coutances (sites 2 and 3). The water temperature was measured manually at each sampling. The data on temperature and precipitation were categorized into three groups whose boundaries were defined so that they are likely to categorize the data significantly for the studied sites and they allow to have in each category a number of sample consistent with a reliable statistical analysis. Comparisons of STEC and EPEC prevalence between the type of samples, the site, the season, the temperature and the precipitation were analyzed by the chi-square test. A *p*-value of <0.05 was considered statistically significant.

## Results

### Detection and Isolation of STEC and EPEC Strains

The *stx* gene was detected in 30.3, 85.9, 41.7, and 28.2% of shellfish, freshwater, seawater and surface sediment enrichment broths, respectively (**Table 1**). The *eae* gene was detected in 74.8, 100, 100, and 43.6% of shellfish, freshwater, seawater, and surface sediment enrichment broths, respectively (**Table 1**). STEC were isolated from 5.0% of the 238 shellfish, 5.6% of the 216 freshwater, 8.3% of the 12 seawater, and 2.6% of the 39 surface sediment samples analyzed, whereas EPEC were isolated from 8.0, 21.3, and 33.3% of the shellfish, freshwater, and seawater samples, respectively. No EPEC were isolated from surface sediments (**Table 2**). A total of 57 STEC and 117 EPEC isolates were obtained from these samples. However, as 29 STEC and 28 EPEC had identical serotypes, PFGE and MLST patterns, virulence gene profiles, and phylogroups to other isolates cultivated from the same samples, they were considered to be replicates and not retained. The remaining 28 STEC and 89 EPEC isolates represented 0.2 and 0.7% of the total *E. coli* (*n* = 12,016), respectively (**Table 3**).

**Table 1:** Prevalence of *stx* and *eae* genes in shellfish, freshwater, seawater, and superficial sediment enrichment broths.

Type of sample	No. of samples	No. of *stx*-positive broth (%)^a^	No. of *eae*-positive broth (%)^a^	No. of *stx*- and *eae*-positive broth (%)^a^
Shellfish	238	72 (30.3)	178 (74.8)	64 (26.9)
Freshwater	216	196 (85.9)	216 (100)	196 (85.9)
Seawater	12	5 (41.7)	12 (100)	5 (41.7)
Superficial sediment	39	11 (28.2)	17 (43.6)	8 (20.5)
**Total**	505	284 (56.2)	423 (83.7)	273 (54.1)

*^a^Percentage calculated based on the total of no. samples for each type of sample.*

**Table 2:** Isolation of STEC and EPEC as regard to sample parameters.

Sample parameter	No. of samples	No. of samples with at least one STEC isolate (%)^a^	No. of samples with at least one EPEC isolate (%)^a^
**Type**			
Shellfish	238	12 (5.0)	19 (8.0)
Freshwater	216	12 (5.6)	46 (21.3)
Seawater	12	1 (8.3)	4 (33.3)
Superficial sediment	39	1 (2.6)	0
χ^2^ test		*p* = 0.845	*p* = 3.09 10^-5^
**Site**			
Site 1	241	10 (4.1)	34 (14.1)
Site 2	157	7 (4.5)	20 (12.7)
Site 3	107	9 (8.4)	15 (14.0)
χ^2^ test		*p* = 0.244	*p* = 0.931
**Season**			
Fall	146	4 (2.7)	11 (7.5)
Summer	126	17 (13.5)	22 (17.5)
Autumn	124	3 (2.4)	24 (19.4)
Winter	109	2 (1.8)	12 (11.0)
χ^2^ test		*p* = 4.44 10^-5^	*p* = 0.0314
**Temperature (°C)**			
0–<10	172	5 (2.9)	15 (8.7)
10–<15	189	14 (7.4)	29 (15.3)
15–>15	144	7 (4.9)	25 (17.4)
χ^2^ test		*p* = 0.167	*p* = 0.086
**Precipitation (mm)**^**b**^			
0–<1	253	17 (6.7)	30 (11.9)
1–<10	144	5 (3.5)	21 (14.6)
10–>10	108	4 (3.7)	18 (16.7)
χ^2^ test		*p* = 0.296	*p* = 0.495
**Total**	505	26 (5.1)	69 (13.7)

*^a^Percentage calculated based on the total of no. samples for each type of sample. ^b^Precipitation: 2-days cumulative rainfall before sampling date.*

**Table 3:** Number of STEC and EPEC strains isolated from shellfish, freshwater, seawater, and superficial sediment samples collected in the three shellfish-harvesting areas and their watersheds, as regard to the total number of *E. coli* isolates.

Type of sample	Total (%)^a^	Shellfish (%)	Freshwater (%)	Seawater (%)	Superficial sediment (%)
**Site 1**					
No. *E. coli* isolates	5,676	1,343	3,410	225	30
No. STEC strains	12 (0.2)	5 (41.7)^b^	6 (50.0)^b^	1 (8.3)^b^	0
No. EPEC strains	47 (0.8)	8 (17.4)^b^	35 (76.1)^b^	4 (8.5)^b^	0
**Site 2**					
No. *E. coli* isolates	3,682	757	2,925	nd^c^	4
No. STEC strains	7 (0.2)	3 (42.9)^b^	4 (57.1)^b^	nd	0
No. EPEC strains	23 (0.6)	10 (43.5)^b^	13 (56.5)^b^	nd	0
**Site 3**					
No. *E. coli* isolates	2,658	678	2,036	nd	83
No. STEC strains	9 (0.3)	4 (44.4)^b^	4 (44.4)^b^	nd	1 (11.1)^b^
No. EPEC strains	19 (0.7)	5 (26.3)^b^	14 (73.7)^b^	nd	0
**Total of the three sites**					
No. *E. coli* isolates	12,016	2,778	8,371	225	117
No. STEC strains	28 (0.2)	12 (42.9)^b^	14 (50.0)^b^	1 (3.6)^b^	1 (3.6)^b^
No. EPEC strains	89 (0.7)	23 (25.8)^b^	62 (69.7)^b^	4 (4.5)^b^	0

*^a^Percentage calculated based on the total of no. *E. coli* isolates from each sites. ^b^Percentage calculated based on the total of no. STEC or EPEC strains isolated from the three sites. ^c^nd: non-determined.*

Shiga-toxin producing *Escherichia coli* strains represented 0.2, 0.2, and 0.3%, of the isolated *E. coli* from sites 1–3, respectively. EPEC strains represented 0.8, 0.6, and 0.7% of the isolated *E. coli* from sites 1–3, respectively (**Table 3**).

For the three sites, the majority of STEC strains derived from freshwater samples (50, 57.1, and 44.4% of samples from sites 1–3, respectively) and from shellfish batches (41.7, 42.9, and 44.4% in the sites 1–3, respectively; **Table 3**). Only two STEC strains were isolated from seawater and surface sediment samples from sites 1 and 3, respectively. The majority of EPEC strains derived from the freshwater samples (76.1, 56.5, and 73.7% of samples from sites 1–3, respectively) and the remaining EPEC derived from shellfish batches (17.4, 43.5, and 26.3%, respectively) and seawater samples (8.5% only in the site 1; **Table 3**).

Nearly one third of the STEC strains were obtained from samples collected in May 2013 (32.1%, 9/28), whereas the EPEC strains were mostly obtained from samples collected in November 2013 (21.3%, 19/89) and August 2014 (12.4%, 11/89; **Table 4**). The entire sample set demonstrated a seasonal effect with potential pathogenic *E. coli* as STEC strains were significantly more frequently isolated in Summer and EPEC strains in Summer and Autumn (*p* < 0.05; **Table 2**). However, no correlation between the prevalence of both STEC and EPEC and pluviometry nor temperature was observed (**Table 2**).

**Table 4:** Characteristics of STEC and EPEC strains isolated from the three French shellfish-harvesting sites from the Eastern English Channel coastal area and their watersheds.

Serotype (no. of isolate)	Virulence gene (no. of isolate)	Sample origin (no. of isolate)	Sampling site (no. of isolate)	Sampling month-year	Precipitation (mm)
**STEC**					
O100:HNM (9)	*stx2*	SFm(1), SFc(1),	2	May-13	0.1
		FW(1), SFo(1), SFm(1)	3	May-13	0.1
		SFm(1), S(1)	3	June-13	6.1
		SFm	2	March-14	0.1
		SFo	3	June-14	0
O15:H16(1)	*stx2*	SFm	1	June-13	0.2
O2:H32(1)	*stx2*	SW	1	February-14	0
O8:H19(2)	*stx2*	FW	3	June-14	0
		FW	3	July-14	1.4
O149:H1(1)	*stx1*	FW	1	April-13	1.6
O149:HNM(1)	*stx1*	FW	1	April-13	1.6
O154:H31(2)	*stx1*	SFo(1), FW(1)	1	May-13	0.1
O154:HNM(1)	*stx1*	SFm	1	November-14	15.4
O28: H1(1)	*stx1*	SFc	1	March-14	0.1
O76:H19(1)	*stx1*	FW	2	August-14	26.0
O88:H25(1)	*stx1*	FW	2	September-14	0.4
ONT:H10(1)	*stx1*	FW	2	April-14	0.3
O26:H11(1)	*stx1+eae+ehxA*	SFm	1	November-13	15.4
O63:H6(1)	*stx2+ehxA+saa*	FW	1	November-14	24.0
ONT:H11(1)	*stx1+stx2+saa*	FW	1	May-13	1.8
O185:H28(1)	*stx1+stx2+ehxA+saa*	FW	2	Apr-13	1.6
O130:H11(1)	*stx1+stx2+ehxA+saa*	FW	1	December-13	0
O91:H21(1)	*stx1+stx2+ehxA+saa*	FW	3	May-13	1.8
**EPEC**					
O103:H25(1)	*eae*	FW	3	March-14	0.1
O103:HNM(1)	*eae*	FW	1	February-14	0
O108:H21(3)	*eae*	FW	3	November-13	0.1
		SFc	2	January-14	6.5
		FW	3	July-14	1.4
O113:H6(4)	*eae*	SFm(1), SFc(1)	1	November-13	15.4
		FW	3	November-13	0.1
		SFo	3	August-14	26
O116:H20(1)	*eae*	FW	1	November-13	15.4
O125:H6(2)	*eae*	FW	1	November-13	15.4
		FW	2	September-14	0.4
O128:H2(1)	*eae*	FW	2	November-13	0
O137:H6(2)	*eae*	FW	1	August-13	1.0
		FW	3	January-15	2.3
O145:H34(1)	*eae*	FW	1	July-14	6.5
O146:H21(1)	*eae*	SFm	3	August-14	26.0
O146:H6(1)	*eae*	SFc	2	September-14	0.4
O15:H2(2)	*eae*	FW	3	August-14	26.0
		SFm	2	January-15	2.3
O153:H21(1)	*eae*	FW	1	January-14	7.2
O157:H16(1)	*eae*	FW	1	July-14	6.5
O159:H7(1)	*eae*	FW	1	November-14	24.0
O167:H3(1)	*eae*	FW	1	August-13	2.8
O179:H31(2)	*eae*	FW	1	January-14	7.2
		FW	3	June-14	0
O2:H45(1)	*eae*	FW	2	December-13	0
O20:HNT(1)	*eae*	SW	1	April-14	0
O23:H8(2)	*eae*	FW	2	August-13	0.7
		SFc	2	August-14	26.0
O25:H2(1)	*eae*	FW	1	September-14	0
O28:H16(1)	*eae*	SFm	1	November-13	15.4
O29:H19(1)	*eae*	FW	2	October-13	22.8
O33:H6(1)	*eae*	FW	1	February-14	0
O39:HNM(1)	*eae*	FW	1	January-14	7.2
O40:HNM(1)	*eae*	FW	1	February-14	0
O42:H37(1)	*eae*	FW	3	March-13	0
O5:H40(1)	*eae*	SFm	2	February-13	0.8
O51:HNM(1)	*eae*	FW	2	October-13	22.8
O63:H6(4)	*eae*	FW	1	November-13	15.4
		FW	2	November-13	0.1
		FW	1	January-14	7.2
		FW	3	October-14	0.5
O63:HNM(1)	*eae*	FW	3	October-14	0.5
O71:H49(2)	*eae*	SFm(1), SFc(1)	1	October-13	0.4
O71:HNM(1)	*eae*	FW	1	August-14	1.0
O8:H14(1)	*eae*	FW	1	November-13	15.4
O85:H31(1)	*eae*	FW	1	October-14	8.3
O85:HNM(1)	*eae*	FW	1	August-14	1.0
O86:H31(1)	*eae*	SFc	2	August-14	26.0
O88:H25(1)	*eae*	SFm	3	September-14	0.4
O88:H8(1)	*eae*	SFo	3	August-14	26.0
O9:HNM(1)	*eae*	FW	2	November-13	0.1
O91:H10(1)	*eae*	SFc	1	July-14	6.5
O98:H56(1)	*eae*	FW	1	December-14	0
O98:H8(1)	*eae*	SFm	1	November-13	15.4
O98:HNM(2)	*eae*	SW	1	February-14	0
		FW	1	March-14	0
O98:HNT(1)	*eae*	FW	1	December-14	0
ONT:H2(1)	*eae*	FW	1	August-13	2.8
ONT:H31(1)	*eae*	FW	1	October-14	8.3
ONT:H34(1)	*eae*	FW	3	December-14	10.6
ONT:H6(6)	*eae*	FW	1	September-13	1.0
		FW	1	August-14	1.0
		FW	3	September-14	0.4
		FW	3	October-14	0.5
		SFm	2	December-14	0
		SW	1	December-14	10.6
ONT:H8(2)	*eae*	FW	1	April-14	0
		FW	1	August-14	1
ONT:HNT(2)	*eae*	SW	1	November-14	24.0
		FW	3	October-14	10.6
O28:HNM(1)	*eae+ehxA*	FW	1	June-14	0.2
O145:H28(2)	*eae+ehxA*	SFm(1), SFc(1)	2	June-13	6.1
O177:H11(1)	*eae+ehxA*	FW	2	July-14	1.4
O26:H11(6)	*eae(5), eae+ehxA(1)*	FW	2	August-13	0.7
		FW(3), SFm(1), SFc(1)	1	November-13	15.4
O103:H2(3)	*eae(2), eae+ehxA(1)*	FW	2	February-13	0.8
		FW	2	June-13	6.1
		FW	1	November-13	15.4
O153:H2(3)	*eae(2), eae+ehxA(2)*	SFm(1), FW(1)	2 (1), 3 (1)	March-14	0.1
		SFc	2	July-14	1.4

*NT, non-typable; NM, non-motile. Sample origin: SF: shellfish, o: oyster, m: mussel, c: cockle, FW: freshwater, SW: seawater, S: superficial sediment.( ): number of strain and when it is not specified the no. of strain is one. (1) Brittany site, (2) first Normandy site, and (3) second Normandy site. Precipitation: 2-days cumulative rainfall before sampling date.*

### Virulence Gene Profiles

By considering the presence of a single virulence gene or a combination of the four virulence genes investigated (i.e., *stx, eae, ehxA*, and *saa*) in the 117 STEC or EPEC strains, eight virulence gene profiles were found. The most frequent profile presented the *eae* gene only (70.1% of the strains) followed by the profile presenting the *stx2* gene only (11.1%) and the profile presenting the *stx1* gene only (7.7%). Seven strains (6.0%) were shown to possess the *eae* and *ehxA* genes. The *stx1-stx2-ehxA-saa* profile was found in three strains and three other virulence gene profiles were observed only once, i.e., the *stx1-eae-ehxA*, the *stx1-stx2-saa*, and the *stx2-ehxA-saa* profiles (**Table 4**).

Seven STEC strains carrying *stx1*, three carrying *stx2*, and two carrying both *stx1* and *stx2* genes were isolated from the site 1 whereas three STEC strains harboring the *stx1* gene, 11 the *stx2* gene, and two presenting both *stx1* and *stx2* genes were recovered from sites 2 and 3.

### Phylogroups

The STEC strains (*n* = 28) were mainly distributed among the phylogroups A, B1, and D (39.3, 35.7, 21.4%, respectively). Only one strain belonged to phylogroup B2. The EPEC strains (*n* = 89) belonged to all the phylogroups; the strains from phylogroup B1 and B2 (38.2 and 38.2%, respectively) being more prevalent than those from phylogroups A and D (18.0 and 5.7%, respectively; **Table 5**). More precisely, at site 1, the STEC strains were mainly divided between phylogroups B1 and D. At sites 2 and 3, the STEC strains were divided between phylogroups A and B1. At sites 1–3, the EPEC strains belonged to all the investigated phylogroups, with a majority belonging to phylogroups B1 and B2.

**Table 5:** Distribution of phylogroup A, B1, B2, and D among STEC and EPEC strains isolated in the three French shellfish-harvesting sites from the Eastern English Channel coastal area and their watersheds.

Phylogroup	Total	A (%)	B1 (%)	B2 (%)	D (%)
**Site 1**					
No. STEC strains (%)^a^	12	2 (16.7)	4 (33.3)	0	6 (50.0)
No. EPEC strains (%)^b^	47	13 (27.7)	13 (27.7)	19 (40.4)	2 (4.4)
**Site 2**					
No. STEC strains (%)^a^	7	3 (42.9)	3 (42.9)	1 (14.2)	0
No. EPEC strains (%)^b^	23	2 (8.7)	13 (56.5)	5 (21.7)	3 (13.0)
**Site 3**					
No. STEC strains (%)^a^	9	6 (66.7)	3 (33.3)	0	0
No. EPEC strains (%)^b^	19	1 (5.3)	8 (42.1)	10 (52.6)	0
**Total of the three sites**					
No. STEC strains (%)^c^	28	11 (39.3)	10 (35.7)	1 (3.6)	6 (21.4)
No. EPEC strains (%)^c^	89	16 (18.0)	34 (38.2)	34 (38.2)	5 (5.7)

*^a^Percentage calculated based on the total no. of STEC strains isolated from each site. ^b^Percentage calculated based on the total no. of EPEC strains isolated from each site. ^c^Percentage calculated based on the total no. of STEC and EPEC strains isolated from the three sites.*

### Serotyping

The 117 (STEC or EPEC) strains selected in this study belonged to 44 O antigens and 24 H antigens and presented 75 distinguishable serotypes (**Table 4**). Among all strains, 13 strains were non-typable (NT) for the O antigen [ONT:H2 (*n* = 1), ONT:H31 (*n* = 1), ONT:H34 (*n* = 1), ONT:H6 (*n* = 6), ONT:H8 (*n* = 2), ONT:H10 (*n* = 1), ONT:H11 (*n* = 1)] and 24 strains were uncharacterized for the H antigen (HNM: non-motile or HNT: non-typable).Two strains were non-typable for either antigens (ONT:HNT).

Eighteen different serotypes (O:H) were identified among the STEC strains. Only one STEC belonging to one of the five highly pathogenic serotypes was isolated: an O26:H11 *stx1^+^, eae^+^*, and *ehxA^+^* strain, from a mussel batch. One strain from serotype O91:H21 and carrying *stx1*, *stx2*, *ehxA*, and *saa* genes was also identified among the STEC strains. The most detected serotype among the STEC strains was the O100:HNM (*n* = 9). Fifteen additional serotypes (O149:H31/HNM, O154:H31/HNM, O130:H11, O15:H16, O185:H28, O2:H32, O28:H1, O63:H11, O76:H19, O8:H12, O88:H25, ONT:H10, and ONT:H11) were identified within the STEC strains and contained one or two individual isolates each.

Fifty-seven serotypes were identified among the EPEC strains. Eleven strains belonged to the highly pathogenic serotypes: O26:H11 (*n* = 6), O103:H2 (*n* = 3), and O145:H28 (*n* = 2). The remaining EPEC strains belong to a large diversity of serotypes listed in **Table 4**.

It should be noted that some serotypes were isolated at different months and in different types of samples. For example, the nine strains of serotype O100:HNM *stx2^+^* were isolated from seven shellfish batches (*n* = 7; oyster, mussel, and common cockle batches), from one freshwater (*n* = 1) and from one surface sediment sample (*n* = 1). Three O154:H31 *stx1^+^* and their immotile form were isolated from two shellfish batches (*n* = 2; oyster and mussel batches) and from one freshwater sample (*n* = 1). The EPEC serotypes, O108:H21 (*n* = 3), O113:H6 (*n* = 4), O15:H2 (*n* = 2), O153:H2 (*n* = 3), O23:H8 (*n* = 2), O26:H11 (*n* = 6), and O71:H49/HNM (*n* = 3) were all isolated from shellfish batches and also from freshwater samples (**Table 4**).

The same serotypes were sometimes isolated from geographically independent sites. For example, serotypes O103:H2, O125:H6, and O26:H11 were isolated from sites 1 and 2 and serotypes O113:H6, O137:H6, and O179:H31 from sites 1 and 3. Finally, the O63:H6 and ONT:H6 serotypes were isolated from all the three sites (**Table 4**).

### PFGE and MLST Profiles

The genetic relatedness of 26 STEC and 79 EPEC strains was investigated by PFGE and MLST analysis (Supplementary Figure S2). Seventy-nine distinguishable PFGE patterns (PT) and 46 distinguishable sequence types (STs) were obtained. Seven other STs (seven strains, one STEC and six EPEC) were obtained but have not as yet been described. These results demonstrate a high level of genetic diversity among the strains isolated. The highest diversity was observed for the EPEC strains, which represented 8.1% of the PTs identified (64/79) and 71.7% of the STs (33/46).

**FIGURE 1:**
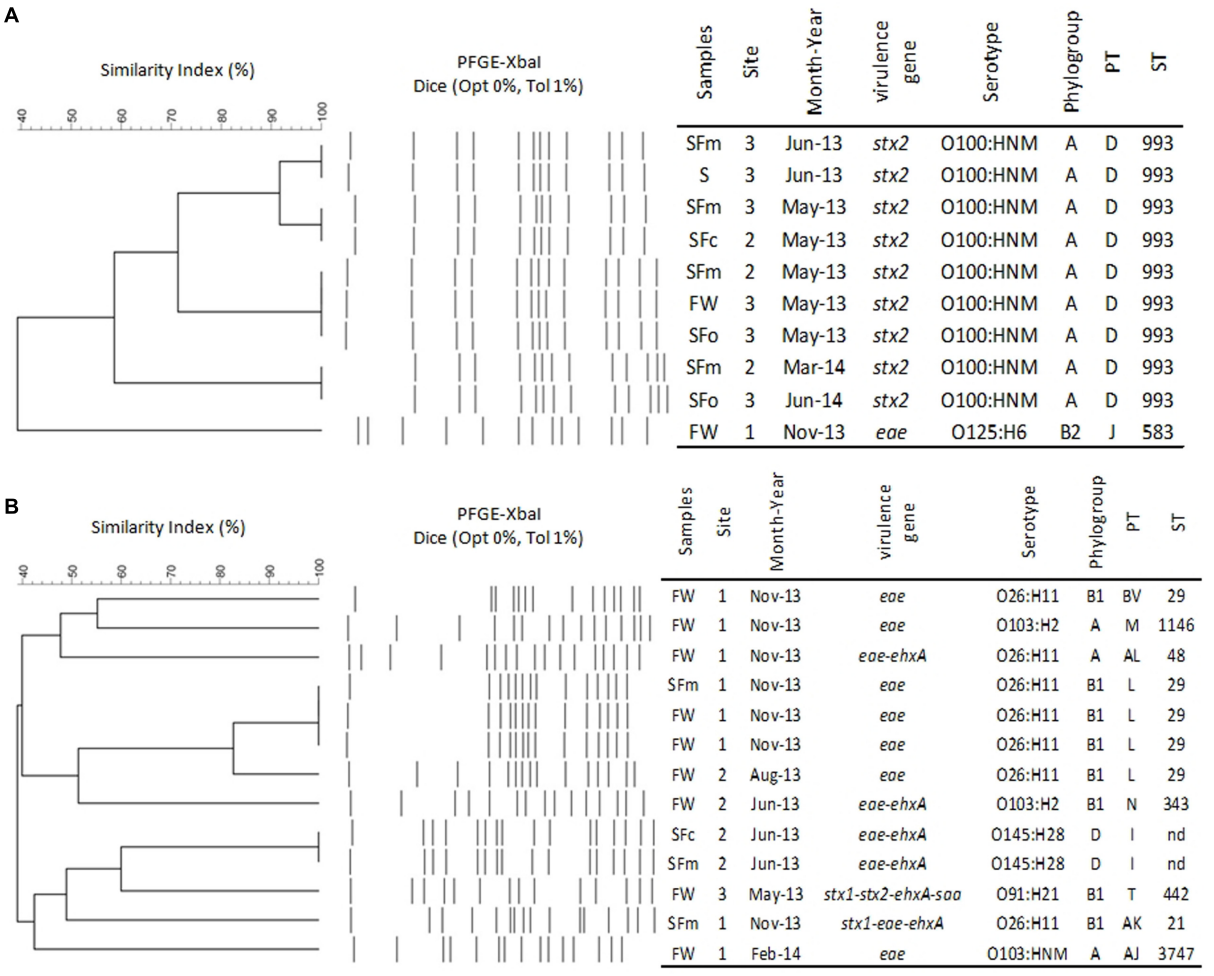
**Focused dendograms of *XbaI* PFGE patterns (PTs), characteristics and Sequence Types (STs) of 9 STEC O100:HNM (A) and the 13 major serotypes (B) isolated from freshwater (FW), shellfish (SF) [oyster (o), mussel (m), or cockle (c)] and superficial sediment (S), from the Brittany site (1) and the two Normandy sites (2 and 3).** The similarity of PFGE profiles was compared and dendogram was created with the Bionumerics software 7.5 (Applied Maths, Belgium), using the band-based Dice unweighted-pair group method, using average linkages (UPGMA), based on 1% position tolerance. NT: non-typable, NM: non-motile, nd: not determined.

The STEC serotype O100:HNM (*n* = 9) presented identical PT (D) and ST (ST933; **Figure 1A**) despite of their three specificities: isolated (a) from oyster, mussel, and common cockle batches, freshwater, and superficial sediment samples, (b) from the sites 2 and 3, (c) during the sampling campaigns of May 2013, June 2013, March 2014, and June 2014. With regards to the major serotypes (**Figure 1B**), the six EPEC O26:H11 strains presented three additional PTs (i.e., L, BV, and AL) and two STs (i.e., ST29 and ST48) isolated from mussel and common cockle batches and freshwater samples. One of those belonging to the ST29 was isolated from the same mussel batch from which the STEC O26:H11 belonging to the ST21 was isolated. A unique PT (I) was observed for the two EPEC O145:H28 (ST not yet described), isolated from mussel and common cockle batches and sampled during the same campaign (June 2013). Both O103:H2 isolated during two different months (i.e., June 2013 and November 2013) had distinguishable PTs (i.e., M and N) and STs (i.e., ST1146 and ST343). Additionally, identical PTs and STs were found among the other STEC and EPEC strains isolated from different types of sample (i.e., shellfish vs. freshwater) or between shellfish batches (i.e., mussel vs. common cockle) and between freshwater samples, often from samples taken from the same sites on the same date (e.g., O153:H2, O108:H21 serotypes; Supplementary Figure S2).

### Biofilm Formation

**FIGURE 2:**
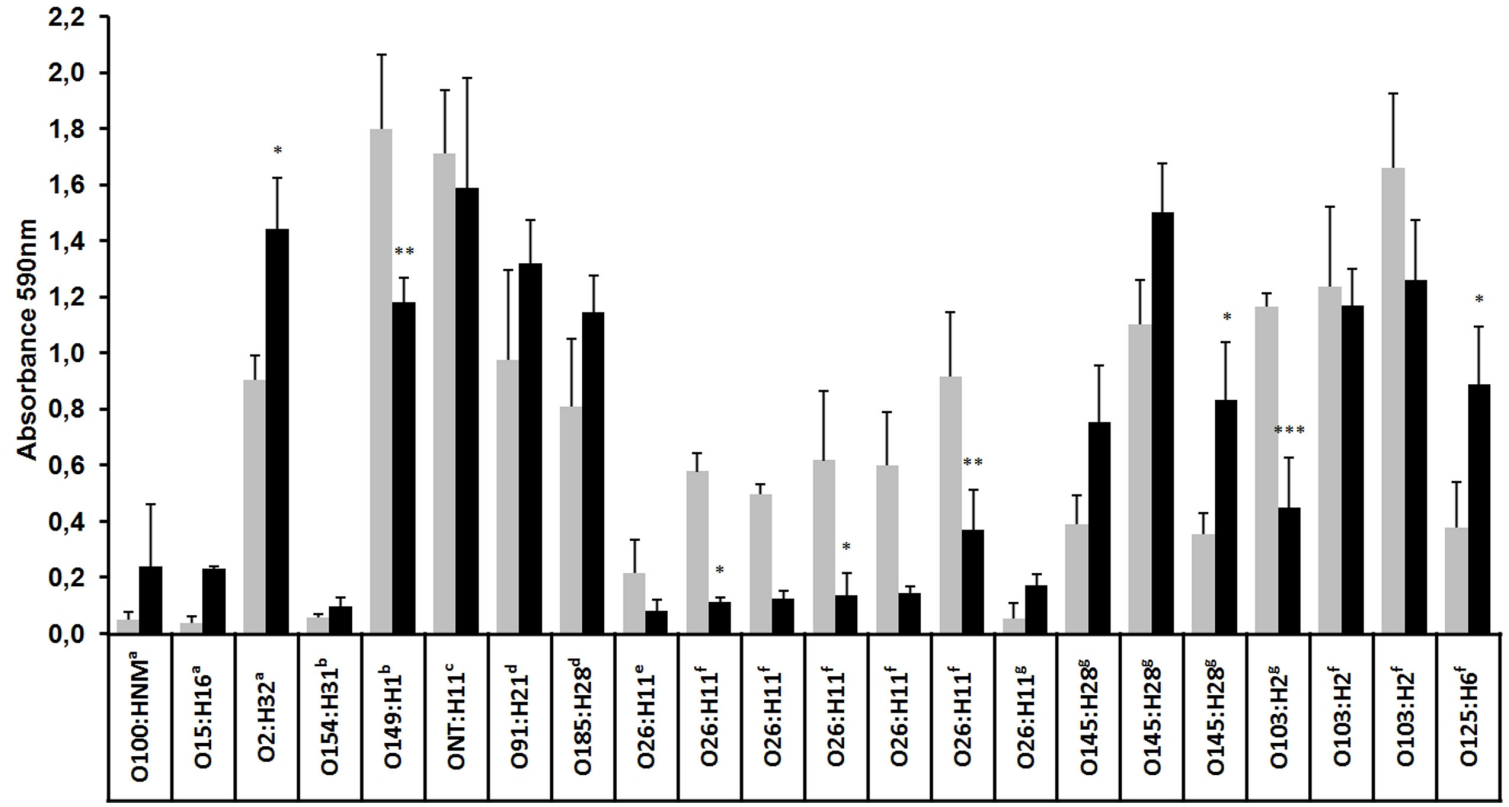
**Biofilm formation by EPEC and STEC strains at low and high temperatures.** Biofilms were formed on polystyrene in M9 medium supplemented with glucose (0.4% wt/vol) at 18°C (gray bars) or 30°C (dark bars) for 24 h and were stained with crystal violet, and the absorbance at 590 nm was measured. The results are the average of at least three biological replicates and the error bar represent the standard error. The results for the static biofilms formed at 18 and 30°C were compared using two-way analysis of variance (ANOVA) followed by a Bonferroni *post hoc* comparison. ^∗^*p* < 0.05; ^∗∗^*p* < 0.01; ^∗∗∗^*p* < 0.001. ^a^*stx2^+^*, ^b^*stx1^+^*, ^c^*stx1^+^-stx2^+^*, ^d^*stx1^+^-stx2^+^-ehxA^+^-saa^+^*, ^e^*stx1^+^-eae^+^*, ^f^*eae^+^*, ^g^*eae^+^-ehxA^+^*.

Biofilm formation by a subset of 13 EPEC and nine STEC strains was evaluated at 18 and 30°C on plastic surface. At both temperatures, strains varied in their ability to form biofilm (OD590 = 0.03 for the lowest, OD590 = 1.9 for the highest). In general, a large number of strains were strongly to moderately adherent and more strains formed a biofilm at 18 than at 30°C (**Figure 2**). Indeed, 11 of the 22 strains formed strong biofilms [serotypes O2:H32, O149:H1, ONT:H11, O91:H21, O185:H28, O26:H11 (*n* = 2), O145:H28 (*n* = 1), O103:H2 (*n* = 3)], six formed medium biofilms [serotypes O26:H11 (*n* = 3), O145:H28 (*n* = 2) and O125:H6] and five formed weak biofilms or no biofilm at all [serotypes O100:HNM, O154:H31, O15:H16, and O26:H11 (*n* = 2)]. At 30°C, 11 strains formed strong biofilms [serotypes O2:H32, O149:H1, ONT:H11, O91:H21, O185:H28, O145:H28 (*n* = 3), O103:H2 (*n* = 2) and O125:H6] but fewer (*n* = 2) were able to form a medium biofilm (O26:H11 and O145:H28) and nine formed a weak biofilm [serotypes O100:HNM, O15:H16, O154:H31, O26:H11 (*n* = 6); **Figure 2**]. Interestingly, all O26:H11 strains formed significantly (Mann–Whitney test) more biofilm (*p* < 0.05) at 18°C than at 30°C.

### Antibiotic Resistance

Antimicrobial susceptibility testing based on the disk diffusion method was performed on 38 EPEC strains and on all STEC strains (*n* = 28). Most of the analyzed STEC and EPEC strains [i.e., 85.7% (24/28) and 73.7% (28/38), respectively] were sensitive toward all 16 used antibiotics. Only four STEC strains were resistant to one antibiotic (i.e., Doxycyclin). With regard to EPEC, 7.9% (3/38) of strains [i.e., serotypes O63:H6, O71:H49, and O98:H8] were resistant to one antibiotic (i.e., Doxycyclin or Imipenem) and 13.2% (5/38) of strains were resistant to two antibiotics (i.e., Cefotaxim/Cefoxitin, O63:H6 or Doxycyclin/Bactrim, O26:H11 or Ticarcillin/Amoxicillin, O26:H11, *n* = 3). One of the O26:H11 serotype was resistant to three antibiotics (i.e., Gentamycin/Ticarcillin/Amoxicillin). Finally, the O2:H45 serotype (*n* = 1) was resistant to four antibiotics (i.e., Cefalotin/Imipenem/Cefotaxim/Cefoxitin).

## Discussion

To our knowledge, this study is the first to focus on the detection and characterization of environmental STEC and EPEC strains from shellfish-harvesting areas and their upstream watershed. Overall, among the environmental samples analyzed (*n* = 505), very few STEC (0.2%, *n* = 28) or EPEC (0.7%, *n* = 89) strains were obtained from the *E. coli* that were isolated (*n* = 12,016) and in comparison with the number of shellfish, water or surface sediment samples that were found to be positive for *stx* and *eae* genes (54.1%, 273/505). The higher proportion of EPEC than STEC strains isolated from these environmental samples is in agreement with the results obtained in previous studies (Hamilton et al., 2010; Chandran and Mazumder, 2015). For example, only 3.6% EPEC and no STEC were detected among the 24,493 *E. coli* isolated from seawater collected in Santa Catalina Island, CA, USA (Hamilton et al., 2010). Conversely, more STEC strains (6.2%) than EPEC strains (1.8%) were isolated from water samples from the Yeongsan river basin in South Korea (*n* = 3,480 *E. coli*; Jang et al., 2014). The low level of isolation of STEC or EPEC vs. the high frequency of detection of genetic markers in the analyzed samples has also been observed in various studies focusing on cattle feces, food, and samples from the environment (Miyagi et al., 2001; Bai et al., 2015; Bibbal et al., 2015). The low level of isolation of STEC vs. the high detection of *stx* genes in the environmental samples could be explained by the presence of free *stx*-encoding bacteriophages in the environment (Martinez-Castillo et al., 2013) and the presence of viable but non-culturable or dead bacteria as a result of the stressful conditions (sunlight, salinity, oligotrophy, predation, etc.) in riverine and especially coastal environments (Gourmelon et al., 1997, for review Rozen and Belkin, 2001). The difficulties of isolating these bacteria from environmental samples containing a significant background flora could also contribute to this low recovery of strains (Pradel et al., 2000; Gourmelon et al., 2006).

The strains isolated in coastal areas of Brittany (site 1) and Normandy (sites 2 and 3) present a high diversity of serotypes as has been previously reported for environmental samples in California or Spain (García-Aljaro et al., 2005; Cooley et al., 2014). A subset of the serotypes isolated in the present study was previously isolated from humans, animals, or the environment. Serotypes such as O8:H14, O26:H11, O76:H19, O91:H21, O103:H2/HNM, O145:H28, and O154:H31 have previously been associated with human infections (Beutin and Fach, 2014). In addition, STEC serotypes O130:H11 and O154:HNM were detected in healthy cattle and waters (Hornitzky et al., 2002), the serotype O157:H16 in dogs, humans, and in the environment (Feng et al., 2012), and the serotype O149:H1 in shellfish (Gourmelon et al., 2006). The STEC O100:HNM was previously detected in swine fecal samples, wild boar feces, and drinking water contaminated by waste water (García-Aljaro et al., 2005; Lienemann et al., 2011; Mora et al., 2012).

In addition, a high genetic diversity among the 105 genotyped strains was observed, with 79 PFGE patterns and 46 distinguishable sequence types in agreement with the high genetic diversity observed by PFGE in other studies for STEC and EPEC strains (Bibbal et al., 2015; Singh et al., 2015). In this study, PFGE was found to be more discriminatory than MLST as previously described for bacteria such as *Salmonella* isolated in Californian coastal waters (Walters et al., 2013). For example, seven strains belonging to ST10 (seven different serotypes) were further discriminated into seven distinct PTs (S, AG, AH, AW, X, G, and Y). The identification of numerous PTs and STs highlights the potential presence of different strains in a same sample and the presence of genetic diversity between strains belonging to the same serotype (e.g., *E. coli* O26:H11 ST21 and ST29 in the same mussel batch).

The non-detection of *E. coli* from the O157:H7 serotype in the shellfish, water and surface sediment samples investigated (from February 2013 to February 2014 (*n* = 282, Balière et al., 2015) is in agreement with the low detection or absence of *E. coli* O157 in shellfish and environmental water previously observed (Miyagi et al., 2001; Manna et al., 2008).

Several EPEC strains belonging to the highly pathogenic serogroups (i.e., O26, O103, and O145) were also isolated from some of the shellfish batches or freshwater samples that were analyzed. The STEC O26:H11 ST21 found in a mussel batch was shown to be implicated in STEC infections and has been detected in cattle in Europe (Zweifel et al., 2013).

The EPEC O26:H11 ST29 isolated in this study can be strains with no previous contact with *stx*-bacteriophages or bacteria that have lost the *stx*-bacteriophage either during their passage from their original source to water or shellfish or during their isolation steps. The presence of these bacteria in coastal environments could present a risk to human health as these EPEC could be lysogenized by *stx1*- or *stx2*-converting bacteriophages, which are present in the same environment and could become STEC of the highly pathogenic serotypes. In fact, Bielaszewska et al. (2007) demonstrated that STEC O26 strains can lose their *stx*-bacteriophages and become EPEC O26, and conversely EPEC O26 can be lysogenized by *stx1*- or *stx2*-bacteriophages and become STEC O26. Even if the conversion of strains was found to occur in the digestive tract of different animals (Toth et al., 2003) and in various food matrices (Imamovic et al., 2009), the environment could also provide the conditions for conversion of strains. However, the potential conversion of *E. coli* strains in the environment still needs to be evaluated in more details (Dumke et al., 2006). Interestingly, Solheim et al. (2013) have demonstrated the conversion of an *E. coli* strain (serotype O103:H25) by bacteriophages in a biofilm at 37°C, but also at 20°C.

In addition, another STEC strain isolated in a freshwater sample could present a potential human risk. Indeed, an O91:H21 *E. coli* was found to belong to ST442, a sequence type that had previously been isolated from adult patients in Germany with symptoms that ranged from diarrhea to hemolytic uremic syndrome (Mellmann et al., 2009).

The majority of strains isolated in this study would present low virulence as most of the isolated strains (87.2%) possessed only one of the five virulence genes (*stx1, stx2, eae, ehxA*, or *saa)*; i.e., 70.1% of the strains carried the *eae* gene, 7.7% *stx1* and 11.1% *stx2*). The STEC O26:H11 was the only STEC isolate to carry the *eae* gene. These results are in agreement with previous studies describing STEC strains isolated from the environment (García-Aljaro et al., 2005) with the exception of the analysis of water samples from a Californian central coast agricultural region where Cooley et al. (2014) showed that the majority of STEC strains isolated contained *stx1*, *stx2*, and *eae* genes. The low level of isolation of STEC strains carrying the *saa* gene encoding another adherence factor, the STEC autoagglutinating adhesion, or the gene *ehxA* encoding enterohemolysin A was in agreement with the results obtained from water samples in Spain by García-Aljaro et al. (2005).

Most of the STEC strains in this study were classified into the A, B1, and D phylogroups. Phylogroups A and B1 were also the main phylogroups of environmental STEC strains isolated by García-Aljaro et al. (2005) in Spain. In the Yeongsan river basin of South Korea, STEC strains isolated belonged mainly to phylogroup D and to a lesser extent to phylogroups A, B1, and B2 (Jang et al., 2014). In Brittany and Normandy, overall, the EPEC strains belonged mainly to phylogroups B1 and B2 and to a lesser extent to A and D. EPEC strains isolated from water samples in South Korea belonged mainly to the B2 phylogroups (Jang et al., 2014). The frequent isolation of *E. coli* from B1 phylogroup in the present study is in agreement with recent data showing that environmentally persistent *E. coli* belong mainly to the B1 phylogroup (Berthe et al., 2013).

Phenotypic differences in the ability to form biofilms among tested strains underline the genetic diversity of STEC or EPEC strains. Our study demonstrates that more than half of the tested strains (17/22) were able to form biofilms on polystyrene at 18 or 30°C, and most of these strains were able to form strong biofilm at 18°C, a temperature close to marine environment condition. A similar result had been observed previously when *E. coli* K12 biofilm was grown at low temperature (White-Ziegler et al., 2008). It has been shown that low temperatures (<30°C) promote the expression of genes associated with biofilm development, including genes involved in curli (*csgA* and *mlrA*) or cellulose (*yaiC*) production (Olsen et al., 1989; Arnqvist et al., 1992; White-Ziegler et al., 2008). Interestingly, although O26:H11 strains formed weaker biofilms than other strains, they formed significantly stronger biofilms at 18°C than at 30°C. Also, all but one O26:H11 strains were negative for *pgaA*, a gene coding for the export of poly-*N*-acetyl-D-glucosamine (PGA) that promotes biofilm formation (Itoh et al., 2008; data not shown). In addition, *pgaA* sequence is also absent in the sequenced genomes of STEC O26 strains available in GenBank. In conclusion, the ability of strains to form a biofilm might contribute to their persistence in coastal environments.

This study highlights the presence of a specific geographic distribution of some of the STEC and EPEC serotypes and a persistence of some of these serotypes in the coastal environments from Brittany and Normandy investigated in this study. The isolation of the serotype O100:HNM positive for *stx2* (PT D and ST993), at different dates (over a period of 1 year), in shellfish, waters and surface sediments from both sites in Normandy (sites 2 and 3), but its absence in Brittany (site 1), highlights potential specific contamination sources in these region and the higher persistence of some of these specific strains. This had previously been shown in water samples from California for O157 strains isolated up to 19 months apart by Cooley et al. (2014). The various livestock breeding in the three watersheds, i.e., mainly swine, poultry and bovine in Brittany and sheep, bovine and swine in Normandy, could explain differences in strain detection at specific sites. A potential explanation of the frequent isolation of the STEC O100:HNM is the high carriage of this *E. coli* strain in the animals in the upstream watersheds. Bibbal et al. (2015) have identified farms harboring STEC bovine carriers, highlighting the act that STEC of a given serotype could be carried by several animals belonging to the same farm. A probable prediction of the presence of these STEC strains in the coastal environment is their re-introduction to the water and consequently to shellfish from animal reservoirs, which enables persistence at high titer for months (Cooley et al., 2014). Another explanation could be that they are present in surface sediments in which a better persistence could occur and then they are re-introduced to the water and then filtered and accumulated by shellfish. The evaluation of the persistence of STEC and EPEC (especially those isolated several times in this study, i.e., *E. coli* O100 and *E. coli* O26) in freshwaters and seawaters and in shellfish needs to be studied to better understand their frequent detection in such shellfish-harvesting areas. Several studies have been carried out to evaluate the persistence of STEC in water or superficial sediments and these have shown that some *E. coli* strains are able to persist in the environment for periods of a few days to several months (Fremaux et al., 2010) and that the persistence could be variable according to the serotypes (Ma et al., 2014).

This study critically evaluated the nature of STEC and EPEC strains present in coastal environments. Knowledge of strains circulating in the environment is crucial to the investigation of potential new STEC serotypes and their human health risk. These results confirm that the environment is a reservoir for these strains. The presence of both EPEC strains and *stx*-converting bacteriophages in the same samples could lead to new pathogenic *E. coli*.

The risk of a human infection by STEC caused by shellfish consumption seems to be limited for two reasons. First, a depuration step or relaying step has to be performed before shellfish from category B and C areas, respectively, reach market. Secondly, STEC were present in only a few samples and the majority of STEC strains lacked genes associated with high human virulence, such as *eae*, and few of the STEC isolated in this study have previously been shown to be involved in human infections.

## Conflict of Interest Statement

The authors declare that the research was conducted in the absence of any commercial or financial relationships that could be construed as a potential conflict of interest.
