# Supplementary material for: Prevalence and Characterization of Shiga Toxin-Producing and Enteropathogenic Escherichia coli in Shellfish-Harvesting Areas and Their Watersheds
Source: Front Microbiol. 2015 Dec 1;6:1356. doi: 10.3389/fmicb.2015.01356 (PMC4664706; doi:10.3389/fmicb.2015.01356)
Supplement: Supplementary file 1 [file Presentation_1.PDF]

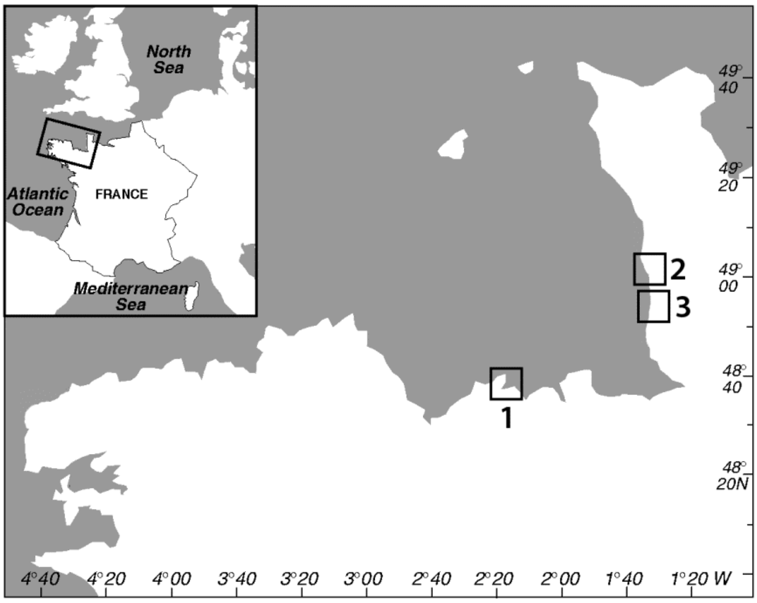

Figure 1S: Map of the three studied shellfish-harvesting sites on the French coast of within the Eastern English Channel. Localization of the Brittany site (site 1) and the two Normandy sites (site 2 and site 3).
