# Supplementary figures and images for: Prevalence and Characterization of Shiga Toxin-Producing and Enteropathogenic Escherichia coli in Shellfish-Harvesting Areas and Their Watersheds
Source: Front Microbiol. 2015 Dec 1;6:1356. doi: 10.3389/fmicb.2015.01356 (PMC4664706; doi:10.3389/fmicb.2015.01356)

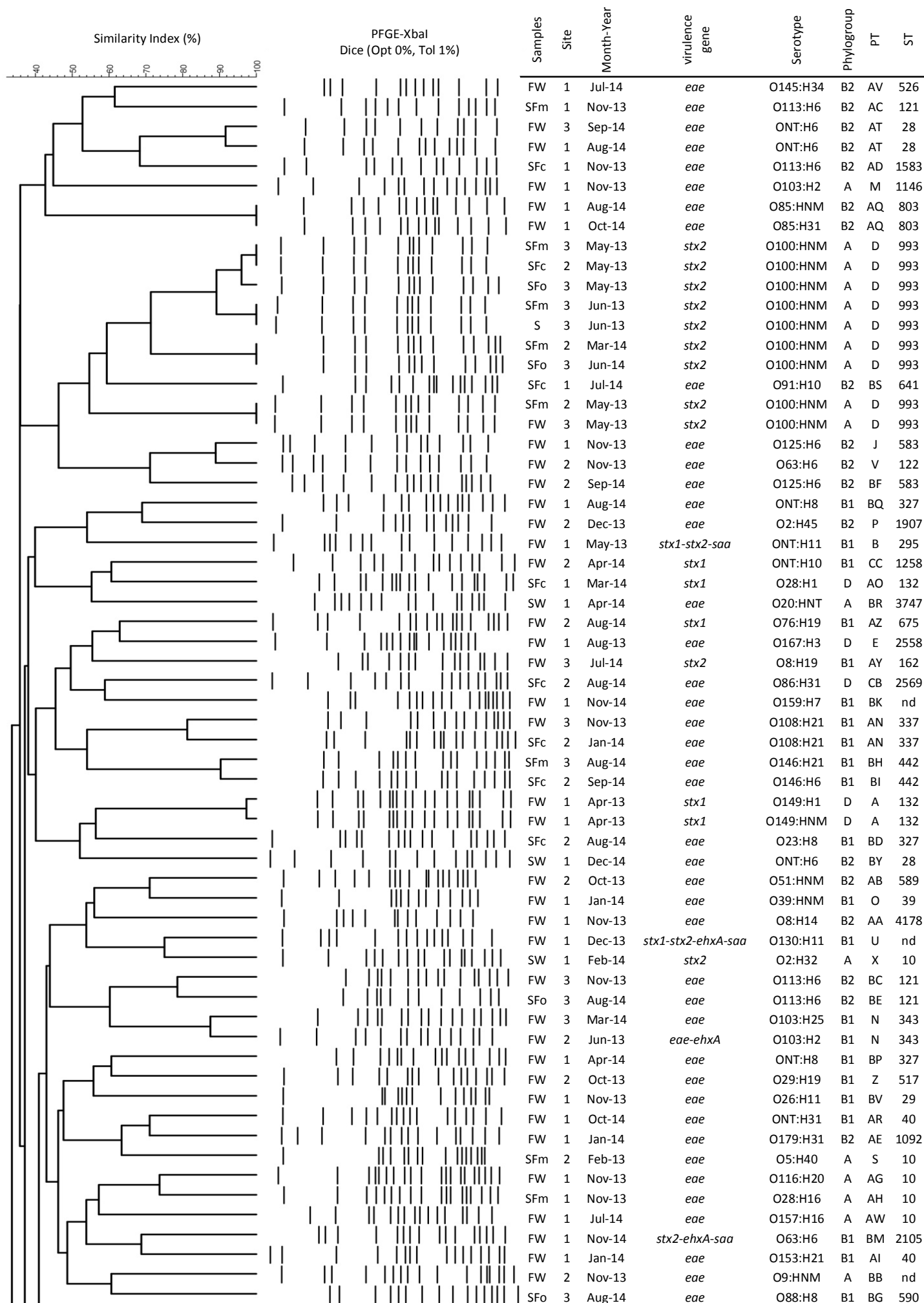

Supplement: Supplementary file 2 [file Presentation_2.PDF]
